# Audit of Local Clinical Governance in London & South Region – 2025

**DOI:** 10.1192/bjo.2025.10660

**Published:** 2025-06-20

**Authors:** Gayathri Rangith, Arokia Antonysamy

**Affiliations:** 1 Cygnet Health Care, Birmingham, United Kingdom; 2 Cygnet Health Care, London, United Kingdom

## Abstract

**Aims:** Clinical governance ensures accountability for continuously improving healthcare quality. This audit evaluates governance compliance across hospital sites in the London & South region, highlighting best practices and opportunities for improvement to enhance patient safety, care standards, and clinical effectiveness.

Aims were to: Identify good clinical governance practices across hospitals to enable peer learning, knowledge sharing and implementation of best practices.

Support continuous improvement by implementing lessons learned from top-performing sites.

**Methods:** Data was collected from Local Clinical Governance meeting minutes (September–December 2024) across multiple hospital sites. Key assessment areas included:

Meeting frequency and leadership involvement.

Attendance and representation from MDT and Operations.

Adherence to governance agenda.

Safety.

Training.

Clinical effectiveness.

Experience.

Leadership.

Audit and research.

Lessons learned.

Standards applied: National Standards on Clinical Audit – NHS England Clinical Governance Framework (2022); Local Clinical governance standards including the STEELL agenda (Safety, Training, Effectiveness, Experience, Leadership, Lessons Learned).

**Results:** Key findings:

Safety and Incident Reporting: Enhanced training programmes contributed to a decline in incidents, across different service lines including Acute, PICU, Rehabilitation, Learning disability and personality disorder units.

Patient and Carer Experience: Positive patient experience achieved with least restrictive practices and removing blanket restrictions with structured feedback from patient councils, advocacy services and Experts by Experience (EbyE).

Clinical Effectiveness and Governance: Higher compliance in care plans and activity programmes were noted in wards with good training and supervision and adherence to clinical models of care

Staffing and Workforce Development: Recruitment strategies helped fill critical vacancies in nursing, psychology, and occupational therapy, ensuring consistent service provision.

Patient Engagement and Activities: Structured activity programmes led to better engagement, particularly where collaborative interdisciplinary teams facilitated therapeutic and skill-based activities.

Areas for Improvement:

Standardisation of digital tracking for patient engagement to ensure accurate compliance data.

Increased MDT participation in governance meetings for enhanced multidisciplinary oversight.

**Conclusion:** Recommendations:

Standardise incident reporting and documentation protocols.

Enhance security for AWOL risk and contraband prevention.

Ensure hospitals share their best practices with the wider group.

**Conclusion:** This audit highlights significant progress in governance, patient engagement, and structured safety interventions across multiple hospital sites. By implementing targeted improvements in data tracking, workforce development, and interdisciplinary collaboration, hospitals can achieve greater compliance, patient-centred care, and long-term service effectiveness. A follow-up audit will assess the impact of these interventions on clinical outcomes and governance excellence.

